# ﻿Seed variability of *Sisymbriumpolymorphum* (Murray) Roth (Brassicaceae) across the Central Palaearctic

**DOI:** 10.3897/phytokeys.206.85673

**Published:** 2022-09-02

**Authors:** Agnieszka Rewicz, Weronika Torbicz, Liudmyla Zavialova, Oksana Kucher, Myroslav V. Shevera, Tomasz Rewicz, Marcin Kiedrzyński, Anna Bomanowska

**Affiliations:** 1 University of Lodz, Department of Geobotany and Plant Ecology, 12/16 Banacha, 90-237 Lodz, Poland National Academy of Sciences of Ukraine Kyiv Ukraine; 2 M.G. Kholodny Institute of Botany, National Academy of Sciences of Ukraine, 2, Tereshchenkivska Str., Kyiv, 01004, Ukraine University of Lodz Lodz Poland; 3 University of Lodz, Department of Invertebrate Zoology and Hydrobiology, 12/16 Banacha, 90-237 Lodz, Poland National Academy of Sciences of Ukraine Kyiv Ukraine; 4 University of Lodz, Department of Biogeography, Palaeoecology and Nature Conservation, Banacha 1/3, 90-237 Lodz, Poland University of Lodz Lodz Poland

**Keywords:** Brassicaceae, latitude variability, micromorphology, scanning electron microscopy (SEM), seed coat, ta­xonomy, 3D ultrastructure

## Abstract

This study presents the results of investigation of the micromorphology and variability of *Sisymbriumpolymorphum* seeds collected in 49 localities in the core range and isolated populations of Central Europe, Eastern Europe and Central Asia. In addition, we compared the ultrastructure of the seeds of S. *polymorphum* with that of the closely-related species *S.loeselii* and *S.linifolium.* The seeds were measured with a stereoscopic microscope and a biometric programme (ImageJ) and micromorphological studies were performed by scanning electron microscopy (SEM). The seed variability showed intraspecific stability of the ultrastructure and low variability of metric features within the studied range. Studied species showed differences in the ultrastructure, which will be valuable for diagnostic purposes. We present and describe, for the first time, the ultrastructure of *S.polymorphum* seeds.

## ﻿Introduction

The differences in the structure of seeds allow for distinguishing individual units at different levels of hierarchy, making them useful in plant taxonomy and identification (Saadaoui et al. 2013; Rewicz et al. 2017, 2020; Martín-Gómez et al. 2019a, 2019b; Ullah et al. 2019). The properties of seeds, such as sculpture variability, are the subject of many scientific studies (Vaughan and Whitehouse 1971; Stace 1993; Gamarra et al. 2010; Kaya et al. 2011; Meyer et al. 2015; Rewicz et al. 2016). Seed shape is crucial in some taxonomic research and, next to sculpture, is one of the most constant characteristics of species (Gamarra et al. 2007; Hadidchi et al. 2020). Moreover, the biometric analysis of individuals from different localities covering a wide range provides objective data on a given taxon’s morphological (as well as habitat and climatic) variability (Skrajna et al. 2012; Rewicz et al. 2017; Kostrakiewicz-Gierałt et al. 2022). Such analyses help to determine the influence of various environmental factors on the phenotypic variability of plants and phylogenetic inference (Bacchetta et al. 2011; Rewicz et al. 2016). Modern imaging methods, for example, scanning electron micro­scopy (SEM), allow for a detailed analysis of seed ultrastructure, relevant in carpological research (Heywood 1969; Hadidchi et al. 2020). The development of new mathematical models and statistical methods also allow for a more precise metric analysis of the tested objects or a more accurate description of shapes (Saadaoui et al. 2013).

The Brassicaceae (Cruciferae) family, commonly known as the mustards, the crucifers or the cabbage family, is widespread worldwide, including 4050 species and 341 genera (Kiefer et al. 2014; Chen et al. 2019). These are primarily herbaceous, annual or perennial plants (Schmidt et al. 2001). Species within the family are centred in regions with a temperate climate; most representatives grow in the Northern Hemisphere (Warwick et al. 2002).

The endo- and exomorphological features of Brassicaceae seeds have been the subject of many taxonomic studies (Musil 1948; Murley 1951; Corner 1976; Koul et al. 2000; Pinar et al. 2007; Karaismailoğlu and Erol 2018; Karaismailoğlu 2019, 2022). Vaughan and Whitehouse (1971) studied the macro- and micromorphology of seeds within 90 genera, including about 200 species of Brassicaceae. Moreover, Kasem et al. (2011) and Antkowiak et al. (2018) paid particular attention to the relationship between the seed structure and the taxonomy of selected species from this family, for example, *Noccacea* spp. and *Thlaspi* spp.

*Sisymbriumpolymorphum* (Murray) Roth is a sub-Irano-Turanian species that occurs in eastern and south-eastern Europe and reaches as far as Mongolia, the vicinity of Irkutsk and Lake Baikal, where it has a diffused range (Kaźmierczakowa and Zarzycki 2001). The western border of its range in Central Europe has some isolated populations in the Pannonian Plateau and the Polish Uplands (Kaźmierczakowa et al. 2014). *Sisymbriumpolymorphum* is currently one of the rarest plants in its westernmost range, probably along with those from the early Holocene migration of steppe plants from the Podolia refuge (Towpasz and Trzcińska-Tacik 1998; Bauer and Somlyay 2007). In Poland, locations are concentrated in the south-eastern part of the Nidziańska Basin in the Małopolska Upland, where relic patches of steppe vegetation have survived (Towpasz and Trzcińska-Tacik 1998). Along with the core part of the range, species vary in abundance: common in the Ukrainian steppe and forest-steppe zones and rare in the Roztochchia-Opillia. It also has some populations in the Polissia and Transcarpathia, where the species can be considered as alien (Ilyinska et al. 2007).

The genus *Sisymbrium* L. (comprising about 50 species) typifies a lack of clear generic boundaries and unique synapomorphies (Al-Shehbaz 1988, 2012, 2015; Warwick et al. 2002; Warwick and Al-Shehbaz 2003; Rūrāne and Roze 2015). The first monograph (Fournier 1865) on the genus listed 166 species, while Schulz (1924), at the beginning of the 20^th^ century, accepted 77 species within the genus and divided it into 14 sections. *Sisymbriumpolymorphum*, together with *S.volgense* M. Bieb. ex E. Fourn., *S.irio* L., *S.austriacum* Jacq. and *S.loeselii* L., were included in The Old World section Irio (Schulz 1924, 1936). Molecular research by Warwick et al. (2002), based on the ITS nuclear region, showed that these species appear in five different terminal clades. The classification proposed by Schulz (1924, 1936) appears utterly incongruent with the above-mentioned molecular studies. *Sisymbriumpolymorphum* appears to be the genetically closest related to *S.loeselii* L. and *S.linifolium* (Nutt.) (Warwick et al. 2002). Further recent studies by Žerdoner Čalasan et al. (2021) involving five nuclear encoded and three chloroplast encoded molecular loci confirm a close relationship with *S.linifolium* and *S.polymorphum*; however, they found *S.polymorphum* paraphyletic or they found some cryptic or pseudo-cryptic species identified as *S.polymorphum* used in their study. Chen et al. (2019) suggest that only molecular identification can help in distinguishing *S.polymorphum* from *S.linifolium.* Our literature review showed a scarcity of data regarding seed size of *S.polymorphum* and a complete lack of information describing the morphological variation of these seeds. Moreover, there is no available information on seed ultrastructure of these closely-related species. Thus, there is no information on whether the genetic closeness of these species is reflected by similarity of the ultrastructure of the seeds.

The presented study aims to: i) analyse differences in the ultrastructure of the seed surface and morphological features of *S.polymorphum* from the core range in Eastern Europe and Central Asia and from isolated localities in Central Europe; ii) indicate differences and similarities between the analysed localities; iii) analysing the variability of the examined morphological traits of seeds and iv) analysing differences in the ultrastructure of the seed surface between *S.polymorphum* and two most closely-related species, *S.loeselii* and *S.linifolium.*

## ﻿Materials and methods

### ﻿Biometric analyses of seeds

**Figure 1.**
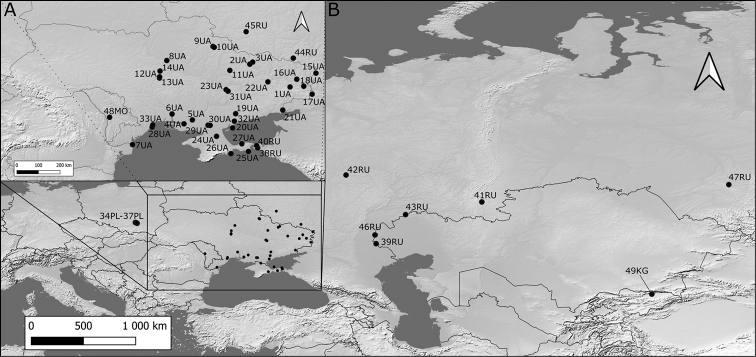
Localities of *Sisymbriumpolymorphum* samples (and zoom window on Ukraine and parts of Russia **(A)** and the rest of the study area **(B**). Codes of localities according to Suppl. material 2: Appendix S1.

We analysed the seeds of *S.polymorphum* (Suppl. material 2: Appendix S1; Fig. 1) from 49 localities. Seeds were loaned out from the following Herbaria: **KRA** (Jagiellonian University), **KW** (M.G. Kholodny Institute of Botany, National Academy of Sciences of Ukraine), **KWHA** (M.M. Gryshko National Botanical Garden, National Academy of Sciences of Ukraine), **MHA** (Main Botanical Garden, Russian Academy of Sciences), **PKM** (Penza I.I. Sprygin Herbarium, Penza State University), **PAV** (Institute of the Eco­logy of the Volga River Basin, Russian Academy of Sciences), **YALT** (Nikitsky Botanical Garden, National Scientific Centre, National Academy of Agrarian Sciences of Ukraine).

For statistical analyses, 20–30 seeds from each locality were measured. We used a Nikon SMZ-800 DS-Fi optical stereomicroscope (Nikon Instruments, Europe B.V.), connected to a Coolview camera 2274 (Nikon) for observation and documentation. To explore the variation in micromorphology, the following features were quantified: seed length (SL), seed width (SW). The terminology follows Bojnanský and Fargašová (2007) and Barthlott et al. (1984).

#### Scanning electron microscopy (SEM)

Micromorphological pictures by scanning electron microscopy were obtained in the facilities: Department of Invertebrate Zoology and Hydrobiology, University of Lodz, Poland (Phenom Pro X) and at the Centre of Electron Microscopy of the M. G. Kholodny Institute of Botany, NAS of Ukraine (JSM-6060 LA). The seeds were fixed on brass tables and were sputter-coated with a 4 nm layer of gold. The SEM photos were taken for 30 tested locations (Suppl. material 2: Appendix S1).

To check if seed ultrastructure showed interspecific differences, we used *Sisymbriumloeselii* seeds from the Herbarium Universitatis Lodziensis (LOD): 085854, 097669, 107817, 153192 and *S.linifolium* from the M.S. Turchaninov memorial collections (KW). The length and width of 25 cells were measured for each species (with the length of the seed). We used SEM pictures (from 18 randomly selected locations) with the same magnification and orientation of the seed.

Three-dimensional models of the ultrastructure of the surface of the seed of *S.polymorphum* were made using 3D Roughness Reconstruction software for the Phenom Electron Microscope.

### ﻿Statistical analyses

The following features were calculated: arithmetic average (x), maximum and minimum values (max and min) and coefficient of variation for traits of *S.polymorphum* (Suppl. material 3: Appendix S2). The ordination of localities according to seeds’ biometric parameters were analysed using a PCA (Principal Components Analysis) scatter plot (van Emden 2019). The relationship between altitude and seed traits for every population was determined with Pearson’s correlation coefficient (r): the following values according to Meissner (2010) were adopted: less than 0.20 = correlation very poor, 0.21–0.39 = weak correlation, 0.40–0.69 = moderate correlation, 0.70–0.89 = strong correlation and above 0.89 = correlation very strong. Cluster analyses showing the morphological similarity of *Sisymbriumpolymorphum* populations according to seed traits were grouped using the UPGMA method.

The Shapiro-Wilk and Kolmogorov-Smirnov tests were conducted to check for a normal distribution of the data; both were not normal; therefore, the Kruskal-Wallis test (for *p* ≤ 0.05) was used as a non-parametric alternative to ANOVA. The post-hoc test (Dunn’s test) was used to show which populations differed significantly by the statistics. The software package STATISTICA PL. ver. 13.1 (Stat-Soft Inc. 2011) was used for all the numerical analyses (van Emden 2019).

### ﻿Description of study species

*Sisymbriumpolymorphum* (Murray) Roth (*Brassicapolymorpha* Murray, *S.junceum* M. Bieb.) is a perennial herb. It grows in grasslands and steppes and disturbed biotopes on the slopes of ravines, gullies, river banks, roadsides, fields and railway embankments. This continental species occurs primarily on gypsum soils, but is currently also encountered on secondarily disturbed biotopes, outcrops or even moderately saline soils (Kaźmierczakowa and Zarzycki 2001; Atlas NBN 2022). The species occurs mainly in lowlands; however, some mountain locations have also been reported. The species’ known altitude range is 300–1900 m a.s.l.

Individuals of *Sisymbriumpolymorphum* grow from 20–80 cm and their leaves are medium in size; scattered-bristly on both sides of the petiole, glabrous above; lower – long-petiolate, broadly lanceolate-pinnately dissected or pinnately dissected, edge from toothed to acute triangular, apex pointed, wedge-shaped base; upper – short-petiolate, narrow, from lanceolate to linear, entire, occasionally unevenly toothed or at the base with two large acute triangular teeth, which turn into elongated lanceolate pointed segments, the base is convergent; succulent-scleromorphic. Stem erect, branched (sometimes from the base), glabrous or below with long straight or twisted protruding hairs. The underground part is a caudex and has a rod-like root system. There are primrose flowers that whiten quickly after flowering. Inflorescence: compound raceme. Pollination: autogamy, entomogamy. Fruit: glabrous pod. Seed dispersal: anemochory and epizoochory. Reproduction can be vegetative (root sprouts) or generative and growth type is unitary and modular (Atlas NBN 2022; WFO 2022).

## ﻿Results

### ﻿Structure of seed surface

The analysis of *S.polymorphum* seeds showed no differences in their ultrastructure between the studied locations. The seeds were glossy and they were orange to brown. The shape of the seeds was observed to be polymorphous from ovate to angular with a subterminal (ST) hilum. The shape of the seeds was not constant within one locality. Pattern sculpture in all studied populations was reticulate and the cells were polygonal in shape (Table 1). We observed that the central part of the seed cells was most often rectangular, while the seeds had a polygonal shape on the sides.

### ﻿Seed size

The analysis of average values of the studied biometric traits of *S.polymorphum* shows that the widest seeds characterise the most northern location in Russia (41, 42RU). The highest average seed width was observed in Asian and Eastern European localities in Russia (average 0.41 mm). The maximum and minimum width values analysis shows that the highest average width occurs in seeds growing in the Russian (42RU) population (average 0.68 mm) (Suppl. material 3: Appendix S2).

Comparatively high values of width are demonstrated in the Polish populations (average width 0.41 mm) and Kyrgyzstan’s (49KG) population (average 0.41 mm). The longest seeds were measured in the Moldovian and Kyrgyzstan populations. The highest averages of length were observed in the following populations: Moldovian (48MO) (average 1.17 mm), as well as Kyrgyzstan’s (49KG) (average 1.03 mm). The maximum values of length (1.43 mm) were recorded in Kyrgyzstan’s (49KG) population (Suppl. material 3: Appendix S2). The minimum values of analysed traits were observed in the Ukrainian populations, with characteristics of the shortest and narrowest seeds in the analysis, with average width 0.36 mm and average length 0.84 mm. The Ukrainian (8UA) population has the smallest seeds both in terms of length (0.49 mm) and width (0.15 mm). The smallest seeds were recorded in the Ukrainian populations and the Russian population had the widest seeds. The features for Polish populations were between those of the other populations. The seeds of *S.polymorphum* displayed a range of variation between all populations.

**Figure 2.**
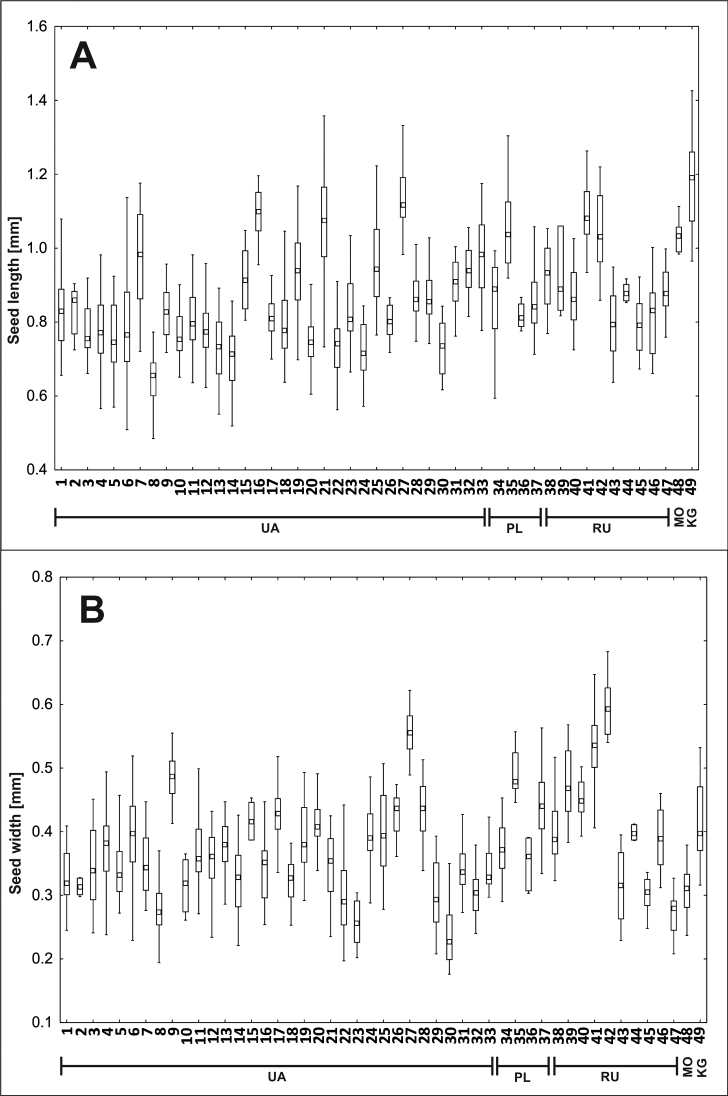
Ranges of variation of seed length **A** and seed width **B** of *Sisymbriumpolymorphum*. The boxes represent the 25^th^–75^th^ percentiles, the upper and lower whiskers extend the minimum and maximum data points and the square inside boxes indicate median. The order of localities is according to Suppl. material 2: Appendix S1.

The studied populations differ significantly in each of the analysed traits, i.e. seed length and width (p < 0.5, Kruskal-Wallis test, Fig. 2, Appendix S3 A, B). The *post-hoc* test showed that, in the case of seed length, the population that differs the most from the others is the population from Ukraine 8UA, which significantly differs statistically from the other 26 populations, mainly from Ukraine, but also from the three populations of Poland (34, 35, 37PL), seven from Russia (38–41, 44, 47RU), the one from Moldova (48MO) and the one from Kyrgyzstan (49KG); the 27UA population was significantly different with 19 populations and the 41RU population was different from 22 populations, mainly from Ukraine. On the other hand, the easternmost population of Kyrgyzstan differs from amongst 23 populations, mainly from Ukraine, but also from one population from Poland (37PL) and two from Russia (43, 45RU).

**Figure 3.**
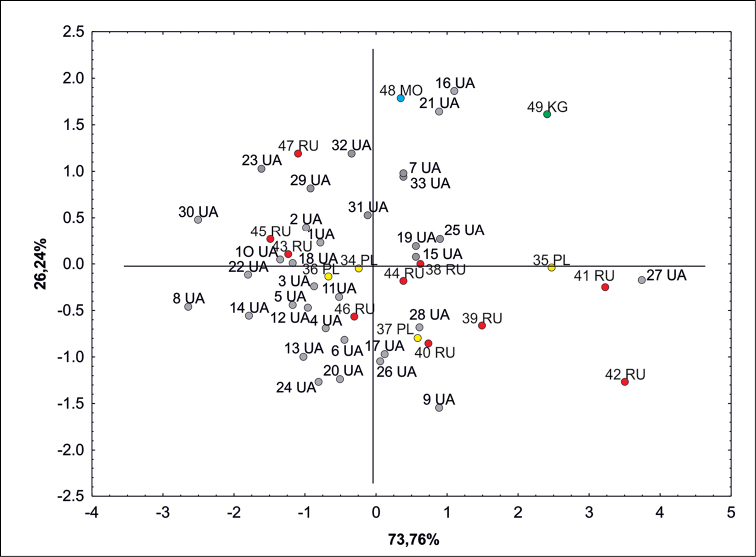
PCA ordination diagram of studied localities of *Sisymbriumpolymorphum* according to the biometric traits of seeds. For codes of populations, see Suppl. material 2: Appendix S1.

Principal Component Analysis (PCA) showed that the studied *S.polymorphum* populations from Russia (41RU, 42RU), Poland (35PL), Ukraine (16UA, 21UA, 27UA), Moldova (48MO) and Kyrgyzstan (49KG) are far from the centre of the PCA ordination space. Still, they cannot be said to form separate groups because the ranges of variability of the examined seed traits coincide (Fig. 3). The first component of PC1 explains 73.76% of the morphological variation, while the second component explains 26.24% of the variation. However, the biogeographical interpretation of both PCA axes is not clear due to the mixing of populations from different parts of the range in the diagram.

**Figure 4.**
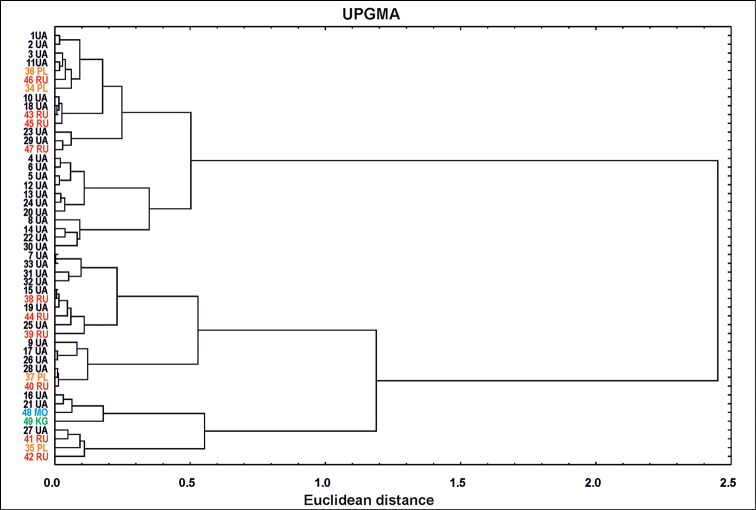
Dendrogram of similarities of *Sisymbriumpolymorphum* populations according to seed traits. For codes of populations, see Suppl. material 2: Appendix S1.

The similarity analysis performed by the cluster analysis showed that only a few Ukrainian populations (4, 5, 6, 8, 12, 13, 14, 20, 22, 24, 30UA) belong to the same cluster (Fig. 4). The analysis of the structure of the remaining clusters does not indicate a homogeneous separation of groups from individual countries or according to the geographical gradient. There is a grouping of populations within one cluster (which is confirmed by the PCA analysis) from Kyrgyzstan (49KG), Moldova (48MO) and particular ones from Russia (41, 42RU), Poland (35PL) and Ukraine (16, 21, 27UA).

**Figure 5.**
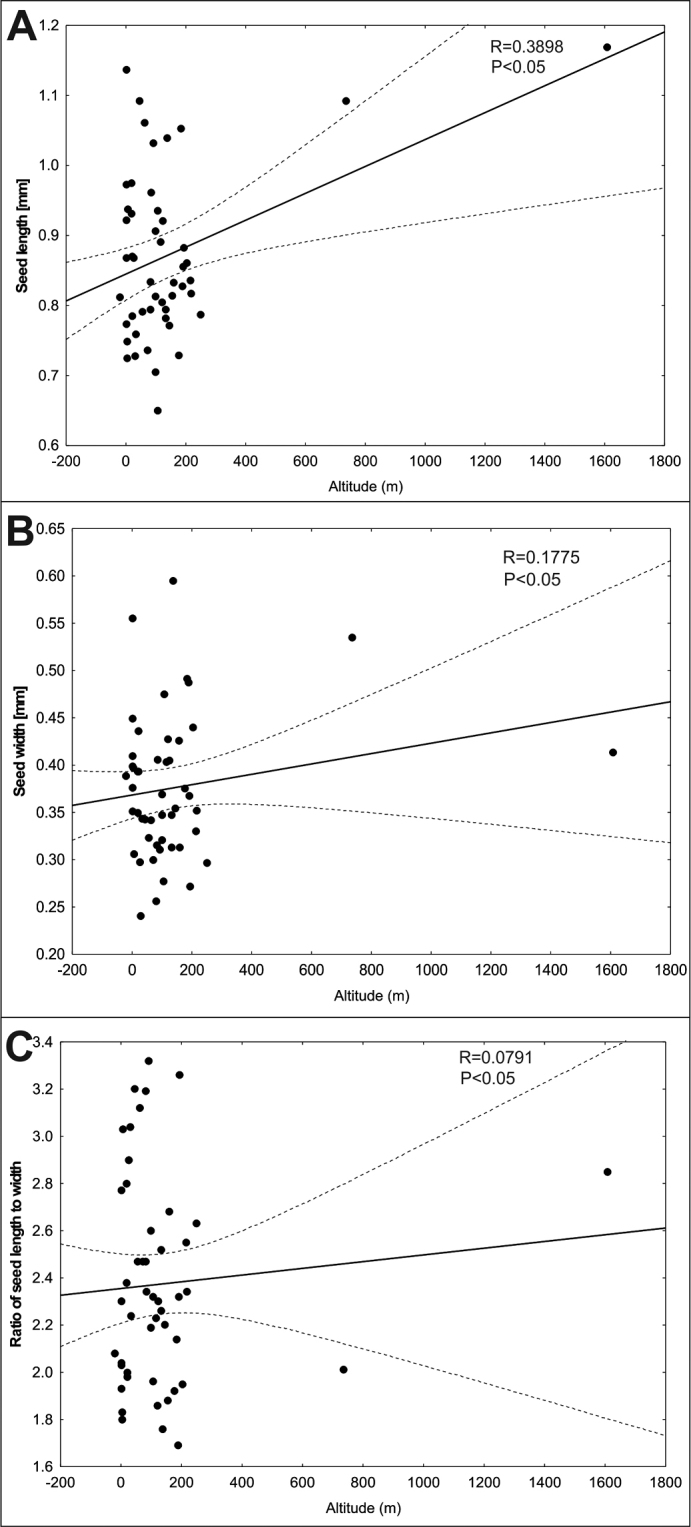
Correlation between **A** seed length **B** seed width **C** ratio of seed length to width of *Sisymbriumpolymorphum* and altitude.

The relationship between the biometric features of seeds and altitude showed a positive, very weak and weak correlation between the studied features. A weak correlation (R = 0.39) (Fig. 5) was demonstrated between seed length and altitude. The weak correlations obtained in our results may be due to the fact that the vast majority of the populations were located at lower altitudes.

### ﻿The structure of seed surface amongst *Sisymbriumpolymorphum*, *S.loeselii* and *S.linifolium*

**Figure 6.**
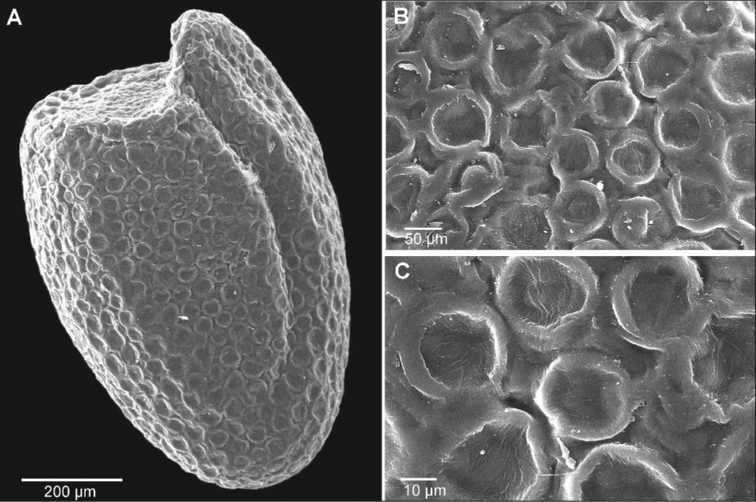
The seed surface of *Sisymbriumlinifolium***A** general view **B** cells **C** ornamentation (sculpture pattern).

**Figure 7.**
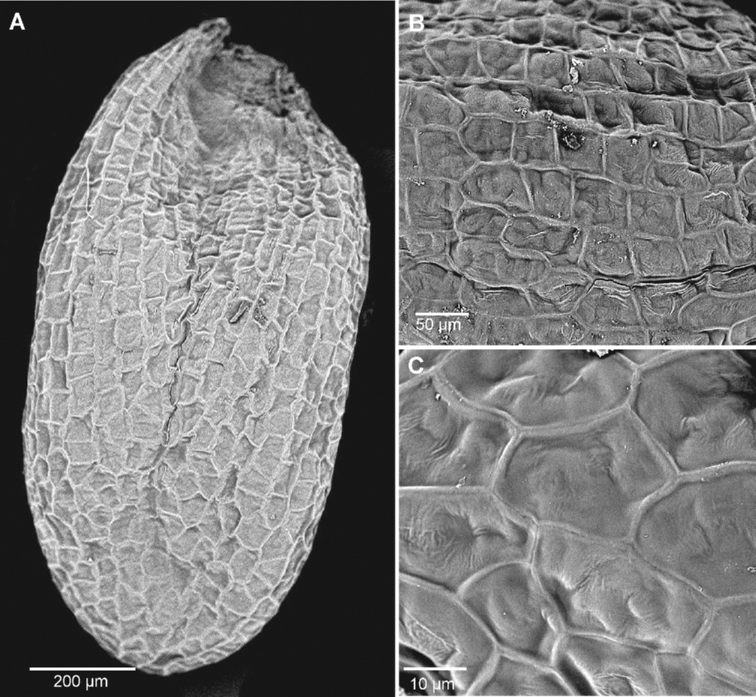
The seed surface of *Sisymbriumpolymorphum***A** general view **B** cells **C** ornamentation (sculpture pattern).

**Figure 8.**
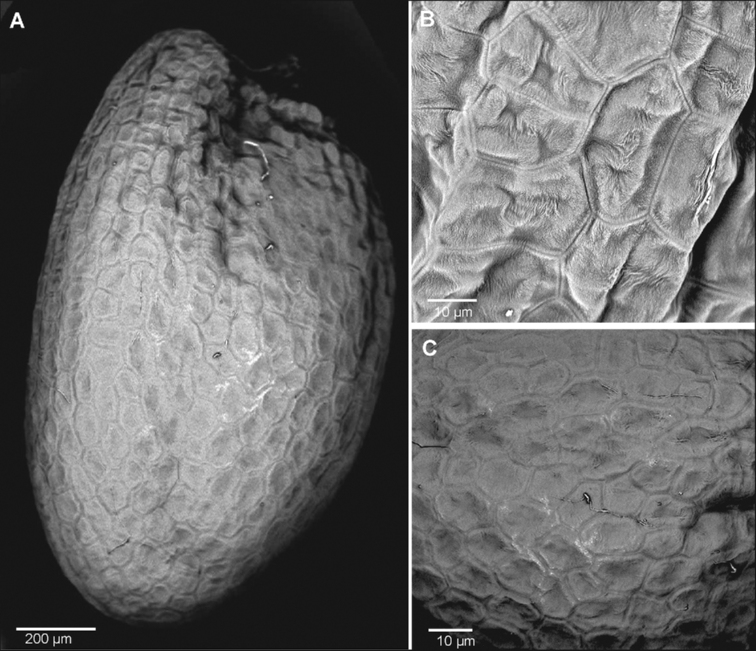
The seed surface of *Sisymbriumloeselii***A** general view **B** cells **C** ornamentation (sculpture pattern).

The seed ultrastructure of the three analysed species showed interspecific differences in ornamentation (Figs 6–8). The species that differed most from the rest was *Sisymbriumlinifolium*. Seeds of *S.linifolium* were characterised by acellate patterns, sculpture and circle shape of cells. In the case of *S.polymorphum* and *S.loeselii* seeds, the ultrastructure was quite similar. Both species had reticulate sculpture patterns and smooth anticlinal and periclinal walls. They differed in the shape of the cells, where it was polygonal in *S.loeselii* and from polygonal to rectangular in *S.polymorphum* (Table 1). The mean length and width of cells of the studied species were the highest in *S.linifolium* (41 μm length × 39 μm width) and *S.polymorphum* (43 μm × 30 μm) (Table 1).

**Table 1.** Seed traits of *S.polymorphum*, *S.loeselii* and *S.linifolium.*

Species	Colour	Shape of seeds	Sculptural pattern	Cell shape	Anticlinal wall	Periclinal wall	Length of cells (μm)	Width of cells (μm)
*S.polymorphum*	orange-brown, shiny	polymorphous from ovate to angular	reticulate	from polygonal to rectangular	smooth	smooth	43	30
*S.loeselii*	orange-brown,	oblong-elipsoid	reticulate	polygonal	smooth	smooth	31	29
*S.linifolium*	light brown	oblong-elipsoid	acellate	circular	smooth	smooth	41	39

## ﻿Discussion

There is a scarcity of literature data on the seed micromorphology of *Sisymbriumpolymorphum* (Sãvulescu et al. 1955; Tutin et al. 1964; Bojňanský and Fargašová 2007; Karpova et al. 2010; Atlas NBN 2022; WFO 2022). Additionally, there seem to be no reports regarding the variability and ultrastructure of the seeds of these plants. Based on previous publications (e.g. Sãvulescu et al. 1955; Tutin et al. 1964; Bojňanský and Fargašová 2007; Atlas NBN 2022; WFO 2022), the length of *S.polymorphum* seeds appears to be within the range 0.6–1.5 mm and the width between 0.3 and 1 mm. The seed dimensions (length, width) recorded in our study are similar to those reported in previous publications (Appendix S2). Our studies indicate lower values in terms of seed minimum length and minimum width (0.49 mm, 0.18 mm, respectively). Moreover, the average seed width (0.24 mm) was lower than reported in previously-published data (0.3–1 mm) (Sãvulescu et al. 1955; Tutin et al. 1964; Bojňanský and Fargašová 2007; www.efloras.org). Our results clearly show statistically significant differences between some populations regarding the analysed features. There were also differences in the coefficient of variation (length WZ 4.14–16.72%, width 4.06–23.93%) (Suppl. material 3: Appendix S2).

Our results did not show significant differences between the core range and isolated populations. However, the longest seeds belonged to the growing population in Kyrgyzstan (49KG) and the Polish populations (37PL), the most westward and second eastward localities. Thus, our research does not confirm that the seeds show variability with longitude change. The multivariate analysis of mean values of seed traits did not show any clear geographic pattern, which can be seen in the PCA analysis (Fig. 3). In our opinion, the size of seeds depends rather on the local habitat and climatic conditions in which a given population grows. Many previous studies (Sultan 2000, 2001; Fried et al. 2008; Skrajna et al. 2013; Szymura and Szymura 2013; Kostrakiewicz-Gierałt et al. 2022) confirm that the main factors affecting the growth and morphological variation of plants include: soil fertility, climate, topographical conditions or type of land use. *S.polymorphum* grows on various types of soil, i.e. rocky, gravel, sandy loams and, sometimes, on saline soils and plaster outcrops. It also occurs in very shallow initial rendzinas soil type made of gypsum in extremely xerothermic loose grasslands (Kaźmierczakowa and Zarzycki 2001). Depending on the region of occurrence, *S.polymorphum* may prefer other conditions. In Poland, localities are associated with gypsum outcrops (Kaźmierczakowa and Zarzycki 2001). There it occurs on steep slopes with inclinations ranging from 5 to 40 (and up to 70) degrees and prefers the top parts. Plants are also reported from gypsum ravines or on top of oblong gypsum hills. They can also appear in anthropogenic habitats: along railway tracks and embankments (Towpasz and Trzcińska-Tacik 1998). In Hungary, they occur mainly on rocky or sandy sites on river banks. However, throughout Eastern Siberia, they prefer sandy, gravel or rocky ground (Towpasz and Trzcińska-Tacik 1998).

The analysed species did not show a high plasticity in terms of shape and colour of the seeds, which is consistent with reports by other authors (Sãvulescu et al. 1955; Gleason and Cronquist 1991; Bojňanský and Fargašová 2007; Cwener and Sudnik-Wojcikowska 2012; www.efloras.org). We observed seeds with polymorphous shapes – from ovate to angular and orange-brown colour. In addition, no variation was observed in the ultrastructure of seeds in the analysed populations. The seeds of the studied species have polygonal to rectangular cells (Suppl. material 1: Fig. S1). The tested seeds showed no variability in ornamentation, which confirms the high stability of this feature. According to Heywood (1974), each genotype has a specific range of plasticity for a given feature, so the analysed feature in some species may show a broader and narrower scale of variation. Knowledge about the plasticity of a given feature allows for the determination of its level and scope of variability and, thus, can indicate whether it might be helpful in the determination of a given species (Heywood 1974; Heywood and Dakshini 1971; Stace 1993). Our research confirms that seed ultrastructure of *S.polymorphum* is stable (has high “resistance” to environmental conditions) and has been shown to provide valuable characters for the delimitation of taxa. The ultrastructure of *S.polymorphum* seeds was here investigated by scanning electron microscopy for the first time.

Our research on the three closely-related *Sisymbrium* species revealed a heterogeneous seed surface structure between these species, the most distinct surface being that of *Sisymbriumlinifolium*. The SEM data (shown in Figs 6–8) indicate that the cell-shaped seed surface (circle) can serve as a suitable diagnostic parameter at the species level within the taxa studied. In the case of the other two species (*S.polymorphum* and *S.loeselii*), the shape of the cells is quite similar (from polygonal to rectangular), which, in our opinion, does not allow for an unequivocal species definition. Chen et al. (2019) could not discriminate specimens of *S.linifolium* and *S.polymorphum* from China morphologically; however, molecular identification, based on ITS nuclear marker data, proved their delimitation as separate species in sister groups. Findings of Žerdoner Čalasan et al. (2021) showed that *S.linifolium* and *S.polymorphum* are phylogenetically closest and *S.loeselii* sister, but also a much wider group containing other species. However, our findings are not contradictory, as morphological features do not always have to follow phylogeny, based on molecular loci (Chaw et al. 1997; Donoghue and Doyle 2000). Our findings appear to support this relationship. There is a clear similarity of these three species, based on seed ultrastructural features. Žerdoner Čalasan et al. (2021) claim that *S.polymorphum* is a highly variable species, both morphologically and genetically. Two samples of *S.polymorphum* analysed in this work were assigned to two different subclades: the easternmost (21748), ca. 800 km SE from 47RU, and the third easternmost (25867), ca. 450 km east from 41RU compared to our data. It may be caused by the occurrence of cryptic (or pseudo-cryptic) species complex or paraphyly. We can also observe many incongruences between results from nuclear and chloroplast loci results. Moreover, Chen et al. (2019) could not discriminate morphologically between *S.linifolium* and *S.polymorphum* and only molecular data were able to prove that the Chinese specimens belonged to *S.linifolium.* Such studies once again demonstrate the importance of interdisciplinary integrative taxonomy. The reticulate type of seed coat is common in other taxa of the Brassicaceae family (Koul et al. 2000; Moazzeni et al. 2007; Karaismailoğlu 2016). Other features of the seed coat surface, such as anticlinal and periclinal cell wall patterns, have been found to be helpful in the delimitation of taxa in Brassicaceae (Vaughan and Whitehouse 1971; Barthlott 1984; Moazzeni et al. 2007; Karaismailoğlu 2016). However, as shown in Figs 6–8, the here investigated species have similar outer periclinal and anticlinal walls, so we could not confirm that they can serve as adequate diagnostic parameters at the species level for the studied taxa. Previous studies have confirmed that seed colour can serve as a diagnostic feature at the generic and specific levels (Kasem et al. 2011; Kaya et al. 2011; Karaismailoğlu 2016, 2019). However, our research does not confirm these reports. Seed colour within the *S.polymorphum* population and between *S.polymorphum*, *S.loeselii* and *S.linifolium* varied from orange to brown to light brown. The addition of this set of micromorphological features to other morphological, biochemical and molecular characters should provide more robust information concerning the phylogenetic affinities between these taxa.

## ﻿Conclusions

Our research proved that, despite the uncleartaxonomic position of *S.polymorphum*, seed ultrastructure is a stable feature and may be useful in taxonomic studies. The study of the seed surface carvings of the other two species shows a difference in the shape and arrangement of cells, especially in the case of *Sisymbriumlinifolium*, which confirms that, in the case of this type of carpological analysis, it can be used in diagnostics. Further integrative studies combining seed ultrastructure, other morphological features and molecular data, may finally solve many taxonomic question marks within the *Sisymbrium* genus.

## Supplementary materials

Supplementary material 1 Figure S1. General view of the seeds (SEM) of *Sisymbriumpolymorphum* populations Data type morphological (.tiff file) Explanation note **Figure S1.** General view of the seeds (SEM) of *Sisymbriumpolymorphum* populations: **A)** 35PL; **B)** 49KG; **C)** 41RU; **D)** 38RU; **E)** 5UA; cells seed 3D; **F)** 10UA; cells seed; **G)** 15UA; H) 21UA; **I)** 17UA; **J)** 34PL. https://binary.pensoft.net/file/738170This dataset is made available under the Open Database License (http://opendatacommons.org/licenses/odbl/1.0/). The Open Database License (ODbL) is a license agreement intended to allow users to freely share, modify, and use this Dataset while maintaining this same freedom for others, provided that the original source and author(s) are credited.Agnieszka Rewicz

Supplementary material 2 Appendix S1. List of studied localities of *Sisymbriumpolymorphum* included in this study Data type occurences (.docx file) Explanation note List of studied localities of *Sisymbriumpolymorphum* included in this study (* - seeds analyzed under SEM). https://binary.pensoft.net/file/738171This dataset is made available under the Open Database License (http://opendatacommons.org/licenses/odbl/1.0/). The Open Database License (ODbL) is a license agreement intended to allow users to freely share, modify, and use this Dataset while maintaining this same freedom for others, provided that the original source and author(s) are credited.Agnieszka Rewicz

Supplementary material 3 Appendix S2. Comparison of biometric traits of *Sisymbriumpolymorphum* seeds in the analysed populations Data type morphological data (.docx file) Explanation note Comparison of biometric traits of *Sisymbriumpolymorphum* seeds in the analysed populations: X arithmetic average; Min, Max maximum and minimum values; SD standard deviation; CV coefficient of variation; SL seed length; SW seed width. https://binary.pensoft.net/file/738172This dataset is made available under the Open Database License (http://opendatacommons.org/licenses/odbl/1.0/). The Open Database License (ODbL) is a license agreement intended to allow users to freely share, modify, and use this Dataset while maintaining this same freedom for others, provided that the original source and author(s) are credited.Agnieszka Rewicz

Supplementary material 4 Appendix S3. Result of the Test Kruskala-Wallisa Test Data type morphological data (.docx file) Explanation note Result of the Kruskal-Wallis Test: A) seed length; B) seed width. https://binary.pensoft.net/file/738173This dataset is made available under the Open Database License (http://opendatacommons.org/licenses/odbl/1.0/). The Open Database License (ODbL) is a license agreement intended to allow users to freely share, modify, and use this Dataset while maintaining this same freedom for others, provided that the original source and author(s) are credited.Agnieszka Rewicz
