# Supplementary figures and images for: ﻿Seed variability of Sisymbriumpolymorphum (Murray) Roth (Brassicaceae) across the Central Palaearctic
Source: PhytoKeys. 2022 Sep 2;206:87–107. doi: 10.3897/phytokeys.206.85673 (PMC9848901; doi:10.3897/phytokeys.206.85673)

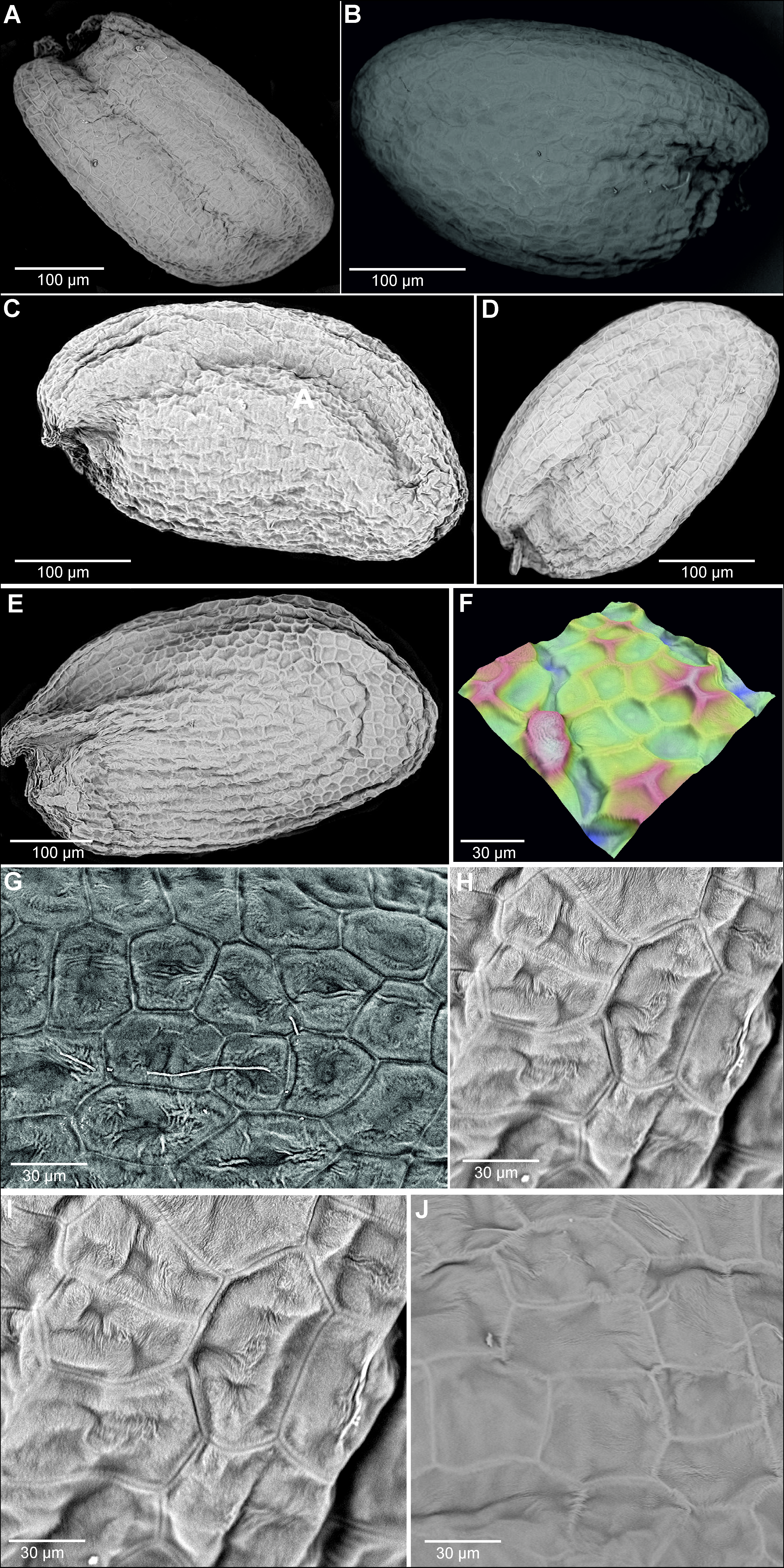

Supplement: Supplementary material 1 — Figure S1. General view of the seeds (SEM) of Sisymbriumpolymorphum populations [file phytokeys-206-087_article-85673__-s001.tif]
