# Supplementary material for: ﻿Seed variability of Sisymbriumpolymorphum (Murray) Roth (Brassicaceae) across the Central Palaearctic
Source: PhytoKeys. 2022 Sep 2;206:87–107. doi: 10.3897/phytokeys.206.85673 (PMC9848901; doi:10.3897/phytokeys.206.85673)
Supplement: Supplementary material 2 — Appendix S1. List of studied localities of Sisymbriumpolymorphum included in this study [file phytokeys-206-087_article-85673__-s002.docx]

**Appendix S1.** List of studied localities of *Sisymbrium polymorphum* included in this study (* - seeds analyzed under SEM)

| **Abbreviation** | **Country** | **Latitude** | **Longitude** | **Altitude** | **Herbarium** |
| --- | --- | --- | --- | --- | --- |
| 1 UA* | Ukraine | 48.54722 | 38.60243 | 215 | KW |
| 2 UA* | Ukraine | 49.79164 | 36.29983 | 159 | KW |
| 3 UA* | Ukraine | 49.90521 | 36.46519 | 133 | KW |
| 4 UA* | Ukraine | 46.53795 | 32.53875 | 1 | KW |
| 5 UA* | Ukraine | 46.75213 | 33.01725 | 33 | KW |
| 6 UA* | Ukraine | 47.07360 | 31.85823 | 22 | KW |
| 7 UA* | Ukraine | 45.40604 | 29.59554 | 1 | KW |
| 8 UA* | Ukraine | 49.99108 | 31.55708 | 106 | KW |
| 9 UA* | Ukraine | 50.75828 | 34.19756 | 189 | KW |
| 10 UA* | Ukraine | 50.71181 | 34.26685 | 132 | KW |
| 11 UA* | Ukraine | 49.45383 | 35.16065 | 100 | KW |
| 12 UA* | Ukraine | 49.03580 | 31.13836 | 144 | KW |
| 13 UA* | Ukraine | 49.10004 | 31.14695 | 177 | KW |
| 14 UA* | Ukraine | 49.41585 | 31.16901 | 100 | KW |
| 15 UA* | Ukraine | 49.30008 | 40.08325 | 124 | KW |
| 16 UA* | Ukraine | 48.97071 | 38.98597 | 45 | KW |
| 17 UA* | Ukraine | 48.15580 | 39.86348 | 156 | KW |
| 18 UA* | Ukraine | 48.58644 | 39.38605 | 56 | KW |
| 19 UA* | Ukraine | 47.08862 | 35.491461 | 19 | KW |
| 20 UA* | Ukraine | 46.31467 | 35.31521 | 3 | KW |
| 21 UA* | Ukraine | 47.28913 | 38.18632 | 63 | KW |
| 22 UA* | Ukraine | 48.82862 | 37.33665 | 72 | KW |
| 23 UA | Ukraine | 48.40298 | 34.94482 | 81 | YALT |
| 24 UA | Ukraine | 45.85653 | 34.39974 | 4 | YALT |
| 25 UA | Ukraine | 45.03383 | 36.22378 | 85 | YALT |
| 26 UA | Ukraine | 44.91358 | 35.22144 | 121 | YALT |
| 27 UA | Ukraine | 45.44772 | 35.84507 | 1 | YALT |
| 28 UA | Ukraine | 46.36385 | 30.72012 | 21 | YALT |
| 29 UA | Ukraine | 46.46358 | 33.91384 | 27 | YALT |
| 30 UA | Ukraine | 46.45412 | 34.03554 | 30 | YALT |
| 31 UA | Ukraine | 48.31797 | 35.05900 | 100 | YALT |
| 32 UA | Ukraine | 46.68205 | 35.41979 | 7 | YALT |
| 33 UA | Ukraine | 46.48258 | 30.75505 | 18 | YALT |
| 34 PL* | Poland | 50.37831 | 20.71629 | 192 | KRA |
| 35 PL* | Poland | 50.46826 | 20.52235 | 184 | KRA |
| 36 PL* | Poland | 50.45287 | 20.58789 | 217 | KRA |
| 37 PL* | Poland | 50.42098 | 20.6721 | 203 | KRA |
| 38 RU* | Russia | 45.22464 | 36.75012 | 2 | KW |
| 39 RU* | Russia | 48.14140 | 46.856280 | 107 | KW |
| 40 RU* | Russia | 45.35175 | 36.69451 | 1 | KW |
| 41 RU* | Russia | 52.72042 | 58.41966 | 735 | KWHA |
| 42 RU | Russia | 55.67828 | 43.56861 | 138 | NNSU |
| 43 RU | Russia | 51.33333 | 50.08333 | 83 | MHA |
| 44 RU | Russia | 50.11981 | 38.78726 | 116 | MHA |
| 45 RU | Russia | 51.56257 | 36.09103 | 250 | MHA |
| 46 RU | Russia | 49.09652 | 46.734075 | -19 | PVB |
| 47 RU | Russia | 54.62249 | 85.43992 | 194 | YALT |
| 48 MO | Moldova | 46.89486 | 28.28720 | 92 | MHA |
| 49 KG* | Kyrgyzstan | 42.60633 | 77.018415 | 1609 | KWHA |
