# Supplementary material for: ﻿Seed variability of Sisymbriumpolymorphum (Murray) Roth (Brassicaceae) across the Central Palaearctic
Source: PhytoKeys. 2022 Sep 2;206:87–107. doi: 10.3897/phytokeys.206.85673 (PMC9848901; doi:10.3897/phytokeys.206.85673)
Supplement: Supplementary material 3 — Appendix S2. Comparison of biometric traits of Sisymbriumpolymorphum seeds in the analysed populations [file phytokeys-206-087_article-85673__-s003.docx]

**Appendix S2.** Comparison of biometric traits of *Sisymbrium polymorphum* seeds in the analyzed populations: X -arithmetic average , Min, Max - maximum and minimum values, SD - standard deviation, CV- coefficient of variation, SL - seed length, SW - the seed width

|  | **Seed width [mm]** | | | | | **Seed length [mm]** | | | | | **Ratio** |
| --- | --- | --- | --- | --- | --- | --- | --- | --- | --- | --- | --- |
| **population code** | **X** | **Min** | **Max** | **SD** | **CV** | **X** | **Min** | **Max** | **SD** | **WZ** | **SL/SW** |
| 1UA | 0.33 | 0.25 | 0.41 | 0.05 | 15.06 | 0.84 | 0.66 | 1.08 | 0.13 | 15.78 | 2.55 |
| 2 UA | 0.31 | 0.30 | 0.33 | 0.01 | 4.06 | 0.83 | 0.73 | 0.90 | 0.07 | 8.46 | 2.68 |
| 3 UA | 0.35 | 0.24 | 0.45 | 0.06 | 18.55 | 0.79 | 0.66 | 1.12 | 0.13 | 16.30 | 2.26 |
| 4 UA | 0.38 | 0.19 | 0.52 | 0.06 | 15.22 | 0.77 | 0.51 | 0.98 | 0.09 | 11.58 | 2.03 |
| 5 UA | 0.34 | 0.27 | 0.49 | 0.05 | 14.44 | 0.76 | 0.57 | 0.92 | 0.09 | 11.91 | 2.24 |
| 6 UA | 0.39 | 0.23 | 0.64 | 0.07 | 17.72 | 0.78 | 0.51 | 1.14 | 0.13 | 16.72 | 2.00 |
| 7 UA | 0.35 | 0.28 | 0.45 | 0.05 | 14.91 | 0.97 | 0.72 | 1.18 | 0.13 | 13.48 | 2.77 |
| 8 UA | 0.28 | 0.15 | 0.46 | 0.05 | 18.50 | 0.65 | 0.49 | 0.83 | 0.07 | 11.48 | 2.32 |
| 9 UA | 0.49 | 0.41 | 0.66 | 0.05 | 9.50 | 0.83 | 0.72 | 0.96 | 0.07 | 8.54 | 1.69 |
| 10 UA | 0.31 | 0.26 | 0.37 | 0.04 | 12.73 | 0.78 | 0.65 | 1.06 | 0.10 | 13.23 | 2.52 |
| 11UA | 0.37 | 0.27 | 0.50 | 0.05 | 12.98 | 0.81 | 0.64 | 0.98 | 0.08 | 9.39 | 2.19 |
| 12 UA | 0.35 | 0.20 | 0.43 | 0.05 | 13.45 | 0.77 | 0.51 | 0.96 | 0.08 | 10.09 | 2.20 |
| 13 UA | 0.38 | 0.22 | 0.45 | 0.05 | 12.49 | 0.73 | 0.55 | 0.89 | 0.09 | 11.81 | 1.92 |
| 14 UA | 0.32 | 0.22 | 0.43 | 0.05 | 16.37 | 0.70 | 0.52 | 0.86 | 0.09 | 13.06 | 2.19 |
| 15 UA | 0.40 | 0.28 | 0.45 | 0.06 | 14.35 | 0.92 | 0.81 | 1.05 | 0.08 | 9.14 | 2.30 |
| 16 UA | 0.34 | 0.25 | 0.45 | 0.05 | 15.04 | 1.09 | 0.96 | 1.20 | 0.07 | 6.65 | 3.20 |
| 17 UA | 0.43 | 0.31 | 0.53 | 0.04 | 9.33 | 0.81 | 0.65 | 0.99 | 0.06 | 7.54 | 1.88 |
| 18 UA | 0.32 | 0.25 | 0.38 | 0.04 | 10.86 | 0.79 | 0.64 | 1.05 | 0.09 | 10.82 | 2.47 |
| 19 UA | 0.39 | 0.29 | 0.49 | 0.06 | 13.99 | 0.93 | 0.70 | 1.17 | 0.11 | 11.77 | 2.38 |
| 20 UA | 0.41 | 0.32 | 0.49 | 0.03 | 8.41 | 0.75 | 0.56 | 1.03 | 0.09 | 11.87 | 1.83 |
| 21 UA | 0.34 | 0.24 | 0.43 | 0.05 | 15.75 | 1.06 | 0.66 | 1.36 | 0.16 | 14.72 | 3.12 |
| 22 UA | 0.30 | 0.20 | 0.44 | 0.06 | 19.54 | 0.74 | 0.56 | 0.91 | 0.08 | 10.97 | 2.47 |
| 23 UA | 0.26 | 0.20 | 0.30 | 0.03 | 12.22 | 0.83 | 0.67 | 1.03 | 0.10 | 11.91 | 3.19 |
| 24 UA | 0.40 | 0.29 | 0.49 | 0.05 | 12.38 | 0.72 | 0.57 | 0.84 | 0.07 | 10.02 | 1.80 |
| 25 UA | 0.41 | 0.28 | 0.64 | 0.08 | 20.94 | 0.96 | 0.77 | 1.22 | 0.12 | 12.96 | 2.34 |
| 26 UA | 0.43 | 0.36 | 0.47 | 0.03 | 7.66 | 0.80 | 0.72 | 0.87 | 0.05 | 5.78 | 1.86 |
| 27 UA | 0.56 | 0.49 | 0.62 | 0.04 | 6.51 | 1.14 | 0.98 | 1.33 | 0.08 | 7.19 | 2.04 |
| 28 UA | 0.44 | 0.34 | 0.51 | 0.05 | 10.69 | 0.87 | 0.75 | 1.01 | 0.07 | 7.67 | 1.98 |
| 29 UA | 0.30 | 0.21 | 0.39 | 0.05 | 16.15 | 0.87 | 0.74 | 1.06 | 0.08 | 8.87 | 2.90 |
| 30 UA | 0.24 | 0.18 | 0.35 | 0.06 | 23.93 | 0.73 | 0.62 | 0.84 | 0.08 | 10.40 | 3.04 |
| 31 UA | 0.35 | 0.27 | 0.47 | 0.04 | 12.77 | 0.91 | 0.76 | 1.00 | 0.07 | 7.48 | 2.60 |
| 32 UA | 0.31 | 0.24 | 0.42 | 0.04 | 13.97 | 0.94 | 0.82 | 1.06 | 0.07 | 7.48 | 3.03 |
| 33 UA | 0.35 | 0.30 | 0.48 | 0.05 | 15.27 | 0.98 | 0.78 | 1.18 | 0.11 | 11.37 | 2.80 |
| 34 PL | 0.37 | 0.22 | 0.45 | 0.05 | 13.18 | 0.86 | 0.59 | 0.99 | 0.12 | 13.59 | 2.32 |
| 35 PL | 0.49 | 0.45 | 0.56 | 0.04 | 7.66 | 1.05 | 0.92 | 1.30 | 0.12 | 11.45 | 2.14 |
| 36 PL | 0.35 | 0.30 | 0.39 | 0.04 | 11.07 | 0.82 | 0.78 | 0.87 | 0.04 | 4.29 | 2.34 |
| 37 PL | 0.44 | 0.29 | 0.59 | 0.06 | 13.85 | 0.86 | 0.71 | 1.10 | 0.09 | 10.12 | 1.95 |
| 38 RU | 0.40 | 0.32 | 0.52 | 0.05 | 12.15 | 0.92 | 0.77 | 1.05 | 0.09 | 9.84 | 2.30 |
| 39 RU | 0.48 | 0.38 | 0.57 | 0.06 | 12.89 | 0.94 | 0.82 | 1.06 | 0.12 | 12.47 | 1.96 |
| 40 RU | 0.45 | 0.39 | 0.50 | 0.03 | 7.01 | 0.87 | 0.73 | 1.03 | 0.09 | 9.84 | 1.93 |
| 41 RU | 0.54 | 0.41 | 0.65 | 0.05 | 9.87 | 1.09 | 0.93 | 1.34 | 0.09 | 8.47 | 2.01 |
| 42 RU | 0.59 | 0.54 | 0.68 | 0.04 | 7.44 | 1.04 | 0.86 | 1.22 | 0.11 | 10.18 | 1.76 |
| 43 RU | 0.32 | 0.23 | 0.40 | 0.06 | 18.44 | 0.79 | 0.64 | 0.95 | 0.11 | 13.33 | 2.47 |
| 44 RU | 0.40 | 0.34 | 0.49 | 0.04 | 10.10 | 0.89 | 0.79 | 1.07 | 0.08 | 8.93 | 2.23 |
| 45 RU | 0.30 | 0.21 | 0.34 | 0.04 | 12.02 | 0.79 | 0.67 | 0.92 | 0.08 | 9.59 | 2.63 |
| 46 RU | 0.39 | 0.31 | 0.46 | 0.05 | 12.09 | 0.81 | 0.66 | 1.00 | 0.10 | 12.85 | 2.08 |
| 47 RU | 0.27 | 0.21 | 0.33 | 0.03 | 11.26 | 0.88 | 0.76 | 1.00 | 0.07 | 7.72 | 3.26 |
| 48 MO | 0.31 | 0.24 | 0.38 | 0.04 | 14.33 | 1.03 | 0.98 | 1.11 | 0.04 | 4.13 | 3.32 |
| 49 KG | 0.41 | 0.32 | 0.53 | 0.07 | 17.02 | 1.17 | 0.97 | 1.43 | 0.13 | 10.89 | 2.85 |
