# Supplementary material for: ﻿Seed variability of Sisymbriumpolymorphum (Murray) Roth (Brassicaceae) across the Central Palaearctic
Source: PhytoKeys. 2022 Sep 2;206:87–107. doi: 10.3897/phytokeys.206.85673 (PMC9848901; doi:10.3897/phytokeys.206.85673)
Supplement: Supplementary material 4 — Appendix S3. Result of the Test Kruskala-Wallisa Test [file phytokeys-206-087_article-85673__-s004.docx]

| **A** | 1UA | 2 UA | 3 UA | 4 UA | 5 UA | 6 UA | 7 UA | 8 UA | 9 UA | 10 UA | 11UA | 12 UA | 13 UA | 14 UA | 15 UA | 16 UA | 17 UA | 18 UA | 19 UA | 20 UA | 21 UA | 22 UA | 23 UA | 24 UA | 25 UA | 26 UA | 27 UA | 28 UA | 29 UA | 30 UA | 31 UA | 32 UA | 33 UA | 34 PL | 35 PL | 36 PL | 37 PL | 38 RU | 39 RU | 40 RU | 41 RU | 42 RU | 43 RU | 44 RU | 45 RU | 46 RU | 47 RU | 48 MO | 49 KG |
| --- | --- | --- | --- | --- | --- | --- | --- | --- | --- | --- | --- | --- | --- | --- | --- | --- | --- | --- | --- | --- | --- | --- | --- | --- | --- | --- | --- | --- | --- | --- | --- | --- | --- | --- | --- | --- | --- | --- | --- | --- | --- | --- | --- | --- | --- | --- | --- | --- | --- |
| 1UA |  |  |  |  |  |  |  | ***** |  |  |  |  |  |  |  |  |  |  |  |  |  |  |  |  |  |  | ***** |  |  |  |  |  |  |  |  |  |  |  |  |  | ***** |  |  |  |  |  |  |  |  |
| 2 UA |  |  |  |  |  |  |  |  |  |  |  |  |  |  |  |  |  |  |  |  |  |  |  |  |  |  |  |  |  |  |  |  |  |  |  |  |  |  |  |  |  |  |  |  |  |  |  |  |  |
| 3 UA |  |  |  |  |  |  |  |  |  |  |  |  |  |  |  | ***** |  |  |  |  | ***** |  |  |  |  |  | ***** |  |  |  |  |  |  |  |  |  |  |  |  |  | ***** | ***** |  |  |  |  |  |  |  |
| 4 UA |  |  |  |  |  |  | ***** | ***** |  |  |  |  |  |  |  | ***** |  |  | ***** |  | ***** |  |  |  | ***** |  | ***** | ***** |  |  | ***** | ***** | ***** |  | ***** |  | ***** | ***** |  |  | ***** | ***** |  |  |  |  | ***** | ***** | ***** |
| 5 UA |  |  |  |  |  |  | ***** |  |  |  |  |  |  |  |  | ***** |  |  | ***** |  | ***** |  |  |  | ***** |  | ***** | ***** |  |  | ***** | ***** | ***** |  | ***** |  | ***** | ***** |  |  | ***** | ***** |  |  |  |  | ***** | ***** | ***** |
| 6 UA |  |  |  |  |  |  | ***** | ***** |  |  |  |  |  |  |  | ***** |  |  | ***** |  | ***** |  |  |  | ***** |  | ***** |  |  |  | ***** | ***** | ***** |  | ***** |  |  | ***** |  |  | ***** | ***** |  |  |  |  |  | ***** | ***** |
| 7 UA |  |  |  |  |  |  |  | ***** |  | ***** | ***** | ***** | ***** |  |  |  | ***** | ***** |  | ***** |  | ***** |  | ***** |  |  |  |  |  | ***** |  |  |  |  |  |  |  |  |  |  |  |  |  |  |  |  |  |  | ***** |
| 8 UA |  |  |  |  |  |  |  |  | ***** |  | ***** | ***** |  |  | ***** | ***** | ***** | ***** | ***** |  | ***** |  | ***** |  | ***** |  | ***** | ***** | ***** |  | ***** | ***** | ***** | ***** | ***** |  | ***** | ***** | ***** | ***** | ***** |  |  | ***** |  |  | ***** | ***** | ***** |
| 9 UA |  |  |  |  |  |  |  |  |  |  |  |  | ***** |  |  | ***** |  |  |  |  | ***** |  |  |  |  |  | ***** |  |  |  |  |  |  |  |  |  |  |  |  |  | ***** |  |  |  |  |  |  |  | ***** |
| 10 UA |  |  |  |  |  |  |  |  |  |  |  |  |  |  |  | ***** |  |  |  |  | ***** |  |  |  | ***** |  | ***** |  |  |  |  | ***** |  |  | ***** |  |  |  |  |  | ***** | ***** |  |  |  |  |  | ***** | ***** |
| 11UA |  |  |  |  |  |  |  |  |  |  |  |  | ***** |  |  | ***** |  |  | ***** |  | ***** |  |  |  | ***** |  | ***** |  |  |  |  | ***** |  |  | ***** |  |  |  |  |  | ***** | ***** |  |  |  |  |  | ***** | ***** |
| 12 UA |  |  |  |  |  |  |  |  |  |  |  |  |  |  |  | ***** |  |  | ***** |  | ***** |  |  |  | ***** |  | ***** | ***** |  |  | ***** | ***** | ***** |  | ***** |  | ***** | ***** |  |  | ***** | ***** |  |  |  |  | ***** | ***** | ***** |
| 13 UA |  |  |  |  |  |  |  |  |  |  |  |  |  |  |  | ***** | ***** |  | ***** |  | ***** |  |  |  | ***** |  | ***** | ***** | ***** |  | ***** | ***** | ***** | ***** | ***** |  | ***** | ***** |  | ***** | ***** | ***** |  |  |  |  | ***** | ***** | ***** |
| 14 UA |  |  |  |  |  |  |  |  |  |  |  |  |  |  |  | ***** |  |  | ***** |  | ***** |  |  |  | ***** |  | ***** | ***** | ***** |  | ***** | ***** | ***** | ***** | ***** |  | ***** | ***** |  | ***** | ***** | ***** |  |  |  |  | ***** | ***** | ***** |
| 15 UA |  |  |  |  |  |  |  |  |  |  |  |  |  |  |  |  |  |  |  |  |  |  |  |  |  |  |  |  |  |  |  |  |  |  |  |  |  |  |  |  |  |  |  |  |  |  |  |  |  |
| 16 UA |  |  |  |  |  |  |  |  |  |  |  |  |  |  |  |  | ***** | ***** |  | ***** |  | ***** |  | ***** |  | ***** |  |  |  | ***** |  |  |  |  |  |  |  |  |  |  |  |  | ***** |  | ***** |  |  |  |  |
| 17 UA |  |  |  |  |  |  |  |  |  |  |  |  |  |  |  |  |  |  | ***** |  | ***** | ***** |  |  | ***** |  | ***** |  |  |  |  | ***** |  |  | ***** |  |  |  |  |  | ***** | ***** |  |  |  |  |  | ***** | ***** |
| 18 UA |  |  |  |  |  |  |  |  |  |  |  |  |  |  |  |  |  |  | ***** |  | ***** |  |  |  | ***** |  | ***** |  |  |  | ***** | ***** | ***** |  | ***** |  |  | ***** |  |  | ***** | ***** |  |  |  |  |  | ***** | ***** |
| 19 UA |  |  |  |  |  |  |  |  |  |  |  |  |  |  |  |  |  |  |  | ***** |  | ***** |  | ***** |  |  |  |  |  | ***** |  |  |  |  |  |  |  |  |  |  |  |  |  |  |  |  |  |  |  |
| 20 UA |  |  |  |  |  |  |  |  |  |  |  |  |  |  |  |  |  |  |  |  | ***** |  |  |  | ***** |  | ***** | ***** | ***** |  | ***** | ***** | ***** |  | ***** |  | ***** | ***** |  |  | ***** | ***** |  |  |  |  | ***** | ***** | ***** |
| 21 UA |  |  |  |  |  |  |  |  |  |  |  |  |  |  |  |  |  |  |  |  |  | ***** |  | ***** |  |  |  |  |  | ***** |  |  |  |  |  |  |  |  |  |  |  |  |  |  | ***** |  |  |  |  |
| 22 UA |  |  |  |  |  |  |  |  |  |  |  |  |  |  |  |  |  |  |  |  |  |  |  |  | ***** |  | ***** | ***** | ***** |  | ***** | ***** | ***** | ***** | ***** |  | ***** | ***** |  | ***** | ***** | ***** |  |  |  |  | ***** | ***** | ***** |
| 23 UA |  |  |  |  |  |  |  |  |  |  |  |  |  |  |  |  |  |  |  |  |  |  |  |  |  |  | ***** |  |  |  |  |  |  |  |  |  |  |  |  |  | ***** |  |  |  |  |  |  |  | ***** |
| 24 UA |  |  |  |  |  |  |  |  |  |  |  |  |  |  |  |  |  |  |  |  |  |  |  |  | ***** |  | ***** | ***** | ***** |  | ***** | ***** | ***** | ***** |  |  | ***** | ***** |  | ***** | ***** | ***** |  |  |  |  | ***** | ***** | ***** |
| 25 UA |  |  |  |  |  |  |  |  |  |  |  |  |  |  |  |  |  |  |  |  |  |  |  |  |  |  |  |  |  | ***** |  |  |  |  |  |  |  |  |  |  |  |  |  |  |  |  |  |  |  |
| 26 UA |  |  |  |  |  |  |  |  |  |  |  |  |  |  |  |  |  |  |  |  |  |  |  |  |  |  | ***** |  |  |  |  |  |  |  |  |  |  |  |  |  | ***** |  |  |  |  |  |  |  | ***** |
| 27 UA |  |  |  |  |  |  |  |  |  |  |  |  |  |  |  |  |  |  |  |  |  |  |  |  |  |  |  | ***** | ***** | ***** |  |  |  | ***** |  |  | ***** |  |  |  |  |  | ***** |  | ***** | ***** |  |  |  |
| 28 UA |  |  |  |  |  |  |  |  |  |  |  |  |  |  |  |  |  |  |  |  |  |  |  |  |  |  |  |  |  |  |  |  |  |  |  |  |  |  |  |  |  |  |  |  |  |  |  |  |  |
| 29 UA |  |  |  |  |  |  |  |  |  |  |  |  |  |  |  |  |  |  |  |  |  |  |  |  |  |  |  |  |  |  |  |  |  |  |  |  |  |  |  |  |  |  |  |  |  |  |  |  |  |
| 30 UA |  |  |  |  |  |  |  |  |  |  |  |  |  |  |  |  |  |  |  |  |  |  |  |  |  |  |  |  |  |  | ***** | ***** | ***** |  | ***** |  |  | ***** |  |  | ***** | ***** |  |  |  |  |  | ***** | ***** |
| 31 UA |  |  |  |  |  |  |  |  |  |  |  |  |  |  |  |  |  |  |  |  |  |  |  |  |  |  |  |  |  |  |  |  |  |  |  |  |  |  |  |  |  |  |  |  |  |  |  |  |  |
| 32 UA |  |  |  |  |  |  |  |  |  |  |  |  |  |  |  |  |  |  |  |  |  |  |  |  |  |  |  |  |  |  |  |  |  |  |  |  |  |  |  |  |  |  |  |  |  |  |  |  |  |
| 33 UA |  |  |  |  |  |  |  |  |  |  |  |  |  |  |  |  |  |  |  |  |  |  |  |  |  |  |  |  |  |  |  |  |  |  |  |  |  |  |  |  |  |  |  |  |  |  |  |  |  |
| 34 PL |  |  |  |  |  |  |  |  |  |  |  |  |  |  |  |  |  |  |  |  |  |  |  |  |  |  |  |  |  |  |  |  |  |  |  |  |  |  |  |  | ***** |  |  |  |  |  |  |  |  |
| 35 PL |  |  |  |  |  |  |  |  |  |  |  |  |  |  |  |  |  |  |  |  |  |  |  |  |  |  |  |  |  |  |  |  |  |  |  |  |  |  |  |  |  |  |  |  |  |  |  |  |  |
| 36 PL |  |  |  |  |  |  |  |  |  |  |  |  |  |  |  |  |  |  |  |  |  |  |  |  |  |  |  |  |  |  |  |  |  |  |  |  |  |  |  |  |  |  |  |  |  |  |  |  |  |
| 37 PL |  |  |  |  |  |  |  |  |  |  |  |  |  |  |  |  |  |  |  |  |  |  |  |  |  |  |  |  |  |  |  |  |  |  |  |  |  |  |  |  | ***** |  |  |  |  |  |  |  | ***** |
| 38 RU |  |  |  |  |  |  |  |  |  |  |  |  |  |  |  |  |  |  |  |  |  |  |  |  |  |  |  |  |  |  |  |  |  |  |  |  |  |  |  |  |  |  |  |  |  |  |  |  |  |
| 39 RU |  |  |  |  |  |  |  |  |  |  |  |  |  |  |  |  |  |  |  |  |  |  |  |  |  |  |  |  |  |  |  |  |  |  |  |  |  |  |  |  |  |  |  |  |  |  |  |  |  |
| 40 RU |  |  |  |  |  |  |  |  |  |  |  |  |  |  |  |  |  |  |  |  |  |  |  |  |  |  |  |  |  |  |  |  |  |  |  |  |  |  |  |  |  |  |  |  |  |  |  |  |  |
| 41 RU |  |  |  |  |  |  |  |  |  |  |  |  |  |  |  |  |  |  |  |  |  |  |  |  |  |  |  |  |  |  |  |  |  |  |  |  |  |  |  |  |  |  | ***** |  | ***** | ***** |  |  |  |
| 42 RU |  |  |  |  |  |  |  |  |  |  |  |  |  |  |  |  |  |  |  |  |  |  |  |  |  |  |  |  |  |  |  |  |  |  |  |  |  |  |  |  |  |  |  |  | ***** |  |  |  |  |
| 43 RU |  |  |  |  |  |  |  |  |  |  |  |  |  |  |  |  |  |  |  |  |  |  |  |  |  |  |  |  |  |  |  |  |  |  |  |  |  |  |  |  |  |  |  |  |  |  |  |  | ***** |
| 44 RU |  |  |  |  |  |  |  |  |  |  |  |  |  |  |  |  |  |  |  |  |  |  |  |  |  |  |  |  |  |  |  |  |  |  |  |  |  |  |  |  |  |  |  |  |  |  |  |  |  |
| 45 RU |  |  |  |  |  |  |  |  |  |  |  |  |  |  |  |  |  |  |  |  |  |  |  |  |  |  |  |  |  |  |  |  |  |  |  |  |  |  |  |  |  |  |  |  |  |  |  |  | ***** |
| 46 RU |  |  |  |  |  |  |  |  |  |  |  |  |  |  |  |  |  |  |  |  |  |  |  |  |  |  |  |  |  |  |  |  |  |  |  |  |  |  |  |  |  |  |  |  |  |  |  |  | ***** |
| 47 RU |  |  |  |  |  |  |  |  |  |  |  |  |  |  |  |  |  |  |  |  |  |  |  |  |  |  |  |  |  |  |  |  |  |  |  |  |  |  |  |  |  |  |  |  |  |  |  |  |  |
| 48 MO |  |  |  |  |  |  |  |  |  |  |  |  |  |  |  |  |  |  |  |  |  |  |  |  |  |  |  |  |  |  |  |  |  |  |  |  |  |  |  |  |  |  |  |  |  |  |  |  |  |
| 49 KG |  |  |  |  |  |  |  |  |  |  |  |  |  |  |  |  |  |  |  |  |  |  |  |  |  |  |  |  |  |  |  |  |  |  |  |  |  |  |  |  |  |  |  |  |  |  |  |  |  |

| **B** | 1UA | 2 UA | 3 UA | 4 UA | 5 UA | 6 UA | 7 UA | 8 UA | 9 UA | 1O UA | 11UA | 12 UA | 13 UA | 14 UA | 15 UA | 16 UA | 17 UA | 18 UA | 19 UA | 20 UA | 21 UA | 22 UA | 23 UA | 24 UA | 25 UA | 26 UA | 27 UA | 28 UA | 29 UA | 30 UA | 31 UA | 32 UA | 33 UA | 34 PL | 35 PL | 36 PL | 37 PL | 38 RU | 39 RU | 40 RU | 41 RU | 42 RU | 43 RU | 44 RU | 45 RU | 46 RU | 47 RU | 48 MO | 49 KG |
| --- | --- | --- | --- | --- | --- | --- | --- | --- | --- | --- | --- | --- | --- | --- | --- | --- | --- | --- | --- | --- | --- | --- | --- | --- | --- | --- | --- | --- | --- | --- | --- | --- | --- | --- | --- | --- | --- | --- | --- | --- | --- | --- | --- | --- | --- | --- | --- | --- | --- |
| 1UA |  |  |  |  |  |  |  |  | * |  |  |  |  |  |  |  | * |  |  |  |  |  |  |  |  |  | * | * |  |  |  |  |  |  | * |  | * |  |  | * | * | * |  |  |  |  |  |  |  |
| 2 UA |  |  |  |  |  |  |  |  | * |  |  |  |  |  |  |  |  |  |  |  |  |  |  |  |  |  | * |  |  |  |  |  |  |  | * |  | * |  |  | * | * | * |  |  |  |  |  |  |  |
| 3 UA |  |  |  |  |  |  |  |  | * |  |  |  |  |  |  |  |  |  |  |  |  |  |  |  |  |  | * |  |  |  |  |  |  |  | * |  |  |  |  |  | * | * |  |  |  |  |  |  |  |
| 4 UA |  |  |  |  |  |  |  | * | * |  |  |  |  |  |  |  | * | * |  |  |  | * | * |  |  |  | * |  | * | * |  | * |  |  |  |  | * |  |  |  | * | * |  |  |  |  | * |  |  |
| 5 UA |  |  |  |  |  |  |  |  | * |  |  |  |  |  |  |  | * |  |  | * |  |  |  |  |  |  | * | * |  |  |  |  |  |  | * |  | * |  |  | * | * | * |  |  |  |  |  |  |  |
| 6 UA |  |  |  |  |  |  |  | * | * |  |  |  |  |  |  |  |  | * |  |  |  | * | * |  |  |  | * |  | * | * |  | * |  |  |  |  |  |  |  |  | * | * |  |  | * |  | * |  |  |
| 7 UA |  |  |  |  |  |  |  |  | * |  |  |  |  |  |  |  | * |  |  |  |  |  |  |  |  |  | * | * |  |  |  |  |  |  | * |  | * |  |  | * | * | * |  |  | * |  |  |  |  |
| 8 UA |  |  |  |  |  |  |  |  | * |  | * | * | * |  | * |  | * |  | * | * |  |  |  | * | * | * | * | * |  |  |  |  |  | * | * |  | * | * | * | * | * | * |  | * |  | * |  |  | * |
| 9 UA |  |  |  |  |  |  |  |  |  | * | * | * | * | * |  | * |  | * | * |  | * | * | * |  | * |  |  |  | * | * | * | * | * | * |  |  |  |  |  |  |  |  | * |  | * |  | * | * |  |
| 1O UA |  |  |  |  |  |  |  |  |  |  |  |  |  |  |  |  | * |  |  | * |  |  |  |  |  | * | * | * |  |  |  |  |  |  | * |  | * |  | * | * | * | * |  |  |  |  |  |  |  |
| 11UA |  |  |  |  |  |  |  |  |  |  |  |  |  |  |  |  | * |  |  |  |  | * | * |  |  |  | * | * | * |  |  |  |  |  | * |  | * |  |  | * | * | * |  |  |  |  | * |  |  |
| 12 UA |  |  |  |  |  |  |  |  |  |  |  |  |  |  |  |  | * |  |  |  |  | * | * |  |  |  | * | * |  |  |  |  |  |  | * |  | * |  |  | * | * | * |  |  |  |  | * |  |  |
| 13 UA |  |  |  |  |  |  |  |  |  |  |  |  |  |  |  |  | * | * |  |  |  | * | * |  |  |  | * |  | * | * |  | * |  |  |  |  | * |  |  |  | * | * |  |  |  |  | * |  |  |
| 14 UA |  |  |  |  |  |  |  |  |  |  |  |  |  |  |  |  | * |  |  | * |  |  |  |  |  | * | * | * |  |  |  |  |  |  | * |  | * |  | * | * | * | * |  |  |  |  |  |  |  |
| 15 UA |  |  |  |  |  |  |  |  |  |  |  |  |  |  |  |  |  |  |  |  |  |  | * |  |  |  |  |  |  |  |  |  |  |  |  |  |  |  |  |  |  |  |  |  |  |  | * |  |  |
| 16 UA |  |  |  |  |  |  |  |  |  |  |  |  |  |  |  |  | * |  |  |  |  |  |  |  |  |  | * |  |  |  |  |  |  |  | * |  | * |  |  | * | * | * |  |  |  |  |  |  |  |
| 17 UA |  |  |  |  |  |  |  |  |  |  |  |  |  |  |  |  |  | * |  |  | * | * | * |  |  |  |  |  | * | * | * | * |  |  |  |  |  |  |  |  |  |  | * |  | * |  | * | * |  |
| 18 UA |  |  |  |  |  |  |  |  |  |  |  |  |  |  |  |  |  |  | * | * |  |  |  | * | * | * | * | * |  |  |  |  |  |  | * |  | * | * | * | * | * | * |  |  |  |  |  |  | * |
| 19 UA |  |  |  |  |  |  |  |  |  |  |  |  |  |  |  |  |  |  |  |  |  | * | * |  |  |  | * |  | * | * |  | * |  |  |  |  |  |  |  |  | * | * |  |  | * |  | * |  |  |
| 20 UA |  |  |  |  |  |  |  |  |  |  |  |  |  |  |  |  |  |  |  |  |  | * | * |  |  |  |  |  | * | * |  | * |  |  |  |  |  |  |  |  |  |  |  |  | * |  | * |  |  |
| 21 UA |  |  |  |  |  |  |  |  |  |  |  |  |  |  |  |  |  |  |  |  |  |  |  |  |  |  | * | * |  |  |  |  |  |  | * |  | * |  |  | * | * | * |  |  |  |  |  |  |  |
| 22 UA |  |  |  |  |  |  |  |  |  |  |  |  |  |  |  |  |  |  |  |  |  |  |  | * | * | * | * | * |  |  |  |  |  | * | * |  | * | * | * | * | * | * |  |  |  |  |  |  | * |
| 23 UA |  |  |  |  |  |  |  |  |  |  |  |  |  |  |  |  |  |  |  |  |  |  |  | * | * | * | * | * |  |  |  |  |  | * | * |  | * | * | * | * | * | * |  | * |  | * |  |  | * |
| 24 UA |  |  |  |  |  |  |  |  |  |  |  |  |  |  |  |  |  |  |  |  |  |  |  |  |  |  | * |  | * | * |  | * |  |  |  |  |  |  |  |  | * |  |  |  | * |  | * |  |  |
| 25 UA |  |  |  |  |  |  |  |  |  |  |  |  |  |  |  |  |  |  |  |  |  |  |  |  |  |  | * |  | * | * |  | * |  |  |  |  |  |  |  |  | * | * |  |  | * |  | * |  |  |
| 26 UA |  |  |  |  |  |  |  |  |  |  |  |  |  |  |  |  |  |  |  |  |  |  |  |  |  |  |  |  | * | * |  | * |  |  |  |  |  |  |  |  |  |  |  |  | * |  | * |  |  |
| 27 UA |  |  |  |  |  |  |  |  |  |  |  |  |  |  |  |  |  |  |  |  |  |  |  |  |  |  |  |  | * | * | * | * | * | * |  | * |  | * |  |  |  |  | * |  | * |  | * | * |  |
| 28 UA |  |  |  |  |  |  |  |  |  |  |  |  |  |  |  |  |  |  |  |  |  |  |  |  |  |  |  |  | * | * | * | * |  |  |  |  |  |  |  |  |  |  | * |  | * |  | * | * |  |
| 29 UA |  |  |  |  |  |  |  |  |  |  |  |  |  |  |  |  |  |  |  |  |  |  |  |  |  |  |  |  |  |  |  |  |  |  | * |  | * | * | * | * | * | * |  |  |  |  |  |  | * |
| 30 UA |  |  |  |  |  |  |  |  |  |  |  |  |  |  |  |  |  |  |  |  |  |  |  |  |  |  |  |  |  |  |  |  |  |  | * |  | * | * | * | * | * | * |  |  |  |  |  |  | * |
| 31 UA |  |  |  |  |  |  |  |  |  |  |  |  |  |  |  |  |  |  |  |  |  |  |  |  |  |  |  |  |  |  |  |  |  |  | * |  | * |  |  | * | * | * |  |  |  |  |  |  |  |
| 32 UA |  |  |  |  |  |  |  |  |  |  |  |  |  |  |  |  |  |  |  |  |  |  |  |  |  |  |  |  |  |  |  |  |  |  | * |  | * | * | * | * | * | * |  |  |  |  |  |  | * |
| 33 UA |  |  |  |  |  |  |  |  |  |  |  |  |  |  |  |  |  |  |  |  |  |  |  |  |  |  |  |  |  |  |  |  |  |  | * |  |  |  |  |  | * | * |  |  |  |  |  |  |  |
| 34 PL |  |  |  |  |  |  |  |  |  |  |  |  |  |  |  |  |  |  |  |  |  |  |  |  |  |  |  |  |  |  |  |  |  |  |  |  |  |  |  |  | * | * |  |  |  |  | * |  |  |
| 35 PL |  |  |  |  |  |  |  |  |  |  |  |  |  |  |  |  |  |  |  |  |  |  |  |  |  |  |  |  |  |  |  |  |  |  |  |  |  |  |  |  |  |  | * |  | * |  | * | * |  |
| 36 PL |  |  |  |  |  |  |  |  |  |  |  |  |  |  |  |  |  |  |  |  |  |  |  |  |  |  |  |  |  |  |  |  |  |  |  |  |  |  |  |  |  |  |  |  |  |  |  |  |  |
| 37 PL |  |  |  |  |  |  |  |  |  |  |  |  |  |  |  |  |  |  |  |  |  |  |  |  |  |  |  |  |  |  |  |  |  |  |  |  |  |  |  |  |  |  | * |  | * |  | * | * |  |
| 38 RU |  |  |  |  |  |  |  |  |  |  |  |  |  |  |  |  |  |  |  |  |  |  |  |  |  |  |  |  |  |  |  |  |  |  |  |  |  |  |  |  | * |  |  |  | * |  | * |  |  |
| 39 RU |  |  |  |  |  |  |  |  |  |  |  |  |  |  |  |  |  |  |  |  |  |  |  |  |  |  |  |  |  |  |  |  |  |  |  |  |  |  |  |  |  |  |  |  | * |  | * |  |  |
| 40 RU |  |  |  |  |  |  |  |  |  |  |  |  |  |  |  |  |  |  |  |  |  |  |  |  |  |  |  |  |  |  |  |  |  |  |  |  |  |  |  |  |  |  | * |  | * |  | * | * |  |
| 41 RU |  |  |  |  |  |  |  |  |  |  |  |  |  |  |  |  |  |  |  |  |  |  |  |  |  |  |  |  |  |  |  |  |  |  |  |  |  |  |  |  |  |  | * |  | * |  | * | * |  |
| 42 RU |  |  |  |  |  |  |  |  |  |  |  |  |  |  |  |  |  |  |  |  |  |  |  |  |  |  |  |  |  |  |  |  |  |  |  |  |  |  |  |  |  |  | * |  | * |  | * | * |  |
| 43 RU |  |  |  |  |  |  |  |  |  |  |  |  |  |  |  |  |  |  |  |  |  |  |  |  |  |  |  |  |  |  |  |  |  |  |  |  |  |  |  |  |  |  |  |  |  |  |  |  |  |
| 44 RU |  |  |  |  |  |  |  |  |  |  |  |  |  |  |  |  |  |  |  |  |  |  |  |  |  |  |  |  |  |  |  |  |  |  |  |  |  |  |  |  |  |  |  |  |  |  | * |  |  |
| 45 RU |  |  |  |  |  |  |  |  |  |  |  |  |  |  |  |  |  |  |  |  |  |  |  |  |  |  |  |  |  |  |  |  |  |  |  |  |  |  |  |  |  |  |  |  |  |  |  |  | * |
| 46 RU |  |  |  |  |  |  |  |  |  |  |  |  |  |  |  |  |  |  |  |  |  |  |  |  |  |  |  |  |  |  |  |  |  |  |  |  |  |  |  |  |  |  |  |  |  |  | * |  |  |
| 47 RU |  |  |  |  |  |  |  |  |  |  |  |  |  |  |  |  |  |  |  |  |  |  |  |  |  |  |  |  |  |  |  |  |  |  |  |  |  |  |  |  |  |  |  |  |  |  |  |  | * |
| 48 MO |  |  |  |  |  |  |  |  |  |  |  |  |  |  |  |  |  |  |  |  |  |  |  |  |  |  |  |  |  |  |  |  |  |  |  |  |  |  |  |  |  |  |  |  |  |  |  |  |  |
| 49 KG |  |  |  |  |  |  |  |  |  |  |  |  |  |  |  |  |  |  |  |  |  |  |  |  |  |  |  |  |  |  |  |  |  |  |  |  |  |  |  |  |  |  |  |  |  |  |  |  |  |
